# Clinical Outcomes of Afatinib Versus Osimertinib in Patients With Non-Small Cell Lung Cancer With Uncommon EGFR Mutations: A Pooled Analysis

**DOI:** 10.1093/oncolo/oyad111

**Published:** 2023-04-28

**Authors:** Chunsheng Wang, Kewei Zhao, Shanliang Hu, Wei Dong, Yan Gong, Conghua Xie

**Affiliations:** Department of Radiation and Medical Oncology, Zhongnan Hospital of Wuhan University, Wuhan, People’s Republic of China; Cancer Center, Union Hospital, Tongji Medical College, Huazhong University of Science and Technology, Wuhan, People’s Republic of China; Department of Radiation Oncology, Yantai Yuhuangding Hospital, Yantai, People’s Republic of China; Department of Radiation Oncology, Yantai Yuhuangding Hospital, Yantai, People’s Republic of China; Department of Biological Repositories, Zhongnan Hospital of Wuhan University, Wuhan, People’s Republic of China; Tumor Precision Diagnosis and Treatment Technology and Translational Medicine, Hubei Engineering Research Center, Zhongnan Hospital of Wuhan University, Wuhan, People’s Republic of China; Department of Radiation and Medical Oncology, Zhongnan Hospital of Wuhan University, Wuhan, People’s Republic of China; Hubei Key Laboratory of Tumor Biological Behaviors, Zhongnan Hospital of Wuhan University, Wuhan, People’s Republic of China; Hubei Cancer Clinical Study Center, Zhongnan Hospital of Wuhan University, Wuhan, People’s Republic of China; Wuhan Research Center for Infectious Diseases and Cancer, Chinese Academy of Medical Sciences, Wuhan, People’s Republic of China

**Keywords:** afatinib, osimertinib, uncommon EGFR mutation, NSCLC

## Abstract

**Background:**

The purpose of this analysis was to investigate the effectiveness of afatinib compared to that of osimertinib in patients with non-small cell lung cancer (NSCLC) who harbored uncommon epidermal growth factor receptor (EGFR) mutations.

**Methods:**

A PubMed database-based literature review was conducted to retrieve related studies. Patients harboring EGFR mutations besides the deletion in exon 19 (19del) and point mutation of L858R were included in this analysis. The primary outcome events were the objective response rate (ORR) and progression-free survival (PFS). Propensity score matching (PSM) at a ratio of 1:1 was used between afatinib and osimertinib groups to control the confounding factors. Uncommon EGFR mutations were categorized into 4 groups: insertion in exon 20 (ex20ins), non-ex20ins single uncommon EGFR mutations, compound EGFR mutations that with 19del or L858R, and compound EGFR mutations without 19del or L858R.

**Results:**

After PSM, 71 patients in either the afatinib or osimertinib group were matched. The afatinib group had an ORR of 60.6%, slightly higher than the osimertinib group’s (50.3%), the difference was not statistically significant (*P* = .610). However, the afatinib group showed a significantly superior PFS benefit than the osimertinib group (11.0 vs. 7.0 months, *P* = .044). In addition, patients harboring non-ex20ins single uncommon EGFR mutations yield the best ORR and PFS, following treatment of either afatinib (ORR: 76.7%, mPFS: 14.1 months) or osimertinib (ORR: 68.8%, mPFS: 15.1 months). Moreover, there was no significant difference in terms of ORR or PFS between the cohort of patients treated with afatinib or osimertinib, regardless of whether or not the patients had brain metastases.

**Conclusions:**

Both afatinib and osimertinib displayed favorable clinical activities toward uncommon EGFR mutations. Afatinib showed a more profound and durable PFS benefit than osimertinib, although no efficacy advantage was observed.

## Teaser

EGFR mutations play an important role in the development and progression of non-small cell lung cancer (NSCLC) This article compares the effectiveness of afatinib and osimertinib in patients with NSCLC who harbored uncommon EGFR mutations.

**Implications for Practice**

Uncommon epidermal growth factor receptor (EGFR) mutations are a heterogeneous population of molecular alterations, and available clinical data on the outcomes of afatinib and osimertinib in patients with non-small cell lung cancer (NSCLC) harboring atypical EGFR mutations is limited. Our research shows that uncommon EGFR mutations exhibit favorable but inconsistent treatment responses and survival outcomes to afatinib and osimertinib. Afatinib showed a more profound and durable PFS benefit than osimertinib, although no efficacy advantage was manifested. Our studies provided a reference for clinicians to formulate individualized and precise treatment plans for patients with NSCLC with uncommon EGFR mutations.

## Introduction

Lung cancer is the deadliest malignancy worldwide. Epidermal growth factor receptor (EGFR) mutations play an important role in both the development and the progression of non-small cell lung cancer (NSCLC).^1,2^ The deletion of exon 19 (19del) as well as the point mutation of L858R are considered to be classical and common EGFR mutations, and account for 80%-90% of all EGFR mutations.^1-3^ In addition, other types of EGFR mutations, known as uncommon EGFR mutations, also have been reported, represent approximately 10%-15% of all EGFR mutations. Those types of EGFR mutations include single uncommon mutations, for example, insertion in exon 19, point mutation of S768I and G719X in exon 18, as well as compound EGFR mutations, like as 19del or L858R compounded with other EGFR mutations, or multiple mutations without 19del or L858R.^4,5^

Most reports on the efficacy of EGFR-TKIs in patients with NSCLC harboring uncommon EGFR mutations are, in some cases, retrospective research with small sample sizes.^6-9^ The post hoc investigation of the LUX-Lung series studies revealed that afatinib (AFA), an irreversible ErbB family blocker, demonstrated clinical activity for the treatment in patients with NSCLC harboring relatively prevalent single uncommon EGFR mutations (G719X, L861Q, and S768I).^8^ Moreover, in the most recent phase II clinical trial, osimertinib (OSI), a third-generation EGFR-TKI, showed favorable anti-tumor action and progression-free survival (PFS) benefit in patients with NSCLC carrying uncommon EGFR mutations.^9^ And it was reported that the pattern of mutations and the presence of partner mutated genes are closely related to the sensitivity of osimertinib to uncommon EGFR mutations.^10,11^

To date, all head-to-head trials comparing the efficacy of different EGFR-TKIs have been conducted in patients with NSCLC with classical EGFR mutations. However, none of them focused specifically on the activity of afatinib and osimertinib in patients who harbor an uncommon EGFR mutation. Therefore, to compare the efficacy of afatinib and osimertinib as well as their effects on PFS in patients harboring uncommon EGFR mutations, we carried out this pooled analysis.

## Methods

### Study Design

An exhaustive literature review was carried out in the PubMed database of the NCBI to search for all of the articles that were pertinent, irrespective of language restriction (updated last on February 28, 2022). The keywords used for this pooled analysis are as follows: rare, uncommon, atypical, mutation, afatinib, osimertinib, EGFR, lung cancer, and NSCLC. In addition, to conduct additional research, we checked thoroughly the reference lists of all relevant articles. The study was undertaken according to the Declaration of Helsinki’s ethical principles and approved by the Ethics Committee of Zhongnan Hospital of Wuhan University. As the work is a publication-based pooled analysis, informed consent was exempted.

### Research Qualifications and Data Collection

A preliminary examination of the searched articles’ titles and abstracts was carried out by 2 authors working in isolation, followed by a second examination of the full-text articles. Articles were included only when they satisfied the following criteria: (1) patients with EGFR mutations apart from 19del as well as L858R (no restrictions on biological resources and detection methods), and they were treated with afatinib or osimertinib; (2) treatment consisted of either afatinib or osimertinib for the patients; (3) studies indicating treatment responses to afatinib or osimertinib; and (4) PFS were disclosed. Data about age, sex, race, smoking history, clinical tumor stage, brain metastasis status, mutation types, responses to afatinib or osimertinib, as well as PFS were all extracted for each qualified study. The objective response rate (ORR) and PFS were clinical endpoint events in this analysis. Response Evaluation Criteria in Solid Tumors (RECIST)-based treatment response assessment was adopted, including complete response (CR), partial response (PR), stable disease (SD), and progressive disease (PD). Where “CR” and “PR” stood for “objective response” (OR). The time between the start of treatment of afatinib or osimertinib and the occurrence of the disease progressing or death was defined as PFS.

### Statistical Analysis

To account for potential confounding variables between patients in the afatinib and osimertinib groups, an optimal propensity score matching (PSM) strategy at a 1:1 ratio was used. The assessments of the propensity score were estimated using logistic regression, which took into account the baseline covariates (age, sex, race, smoking background, tumor stage, brain metastasis status, number of mutations, and treatment lines of AFA/OSI). Fisher’s exact or chi-square test was applied to compare the baseline characteristics of the 2 groups before and after matching. Chi-square analysis was performed to compare the efficacy, and the odds ratio (Ora) and 95% confidence interval (CI) were measured using a logistic regression approach. The Kaplan-Meier method (log-rank test) was used to estimate PFS, and hazard ratio (HR), as well as 95% CI, were determined by using Cox proportional hazards model. ORR and PFS were compared using logistic regression and the Cox proportional hazards models, respectively, in subgroup patients classified by baseline variables. SPSS 22.0 (Chicago, USA) was used for the analysis, and *P* < .05 (2-tailed) was regarded as statistically significant.

## Results

### Search Results

**Figure 1.**
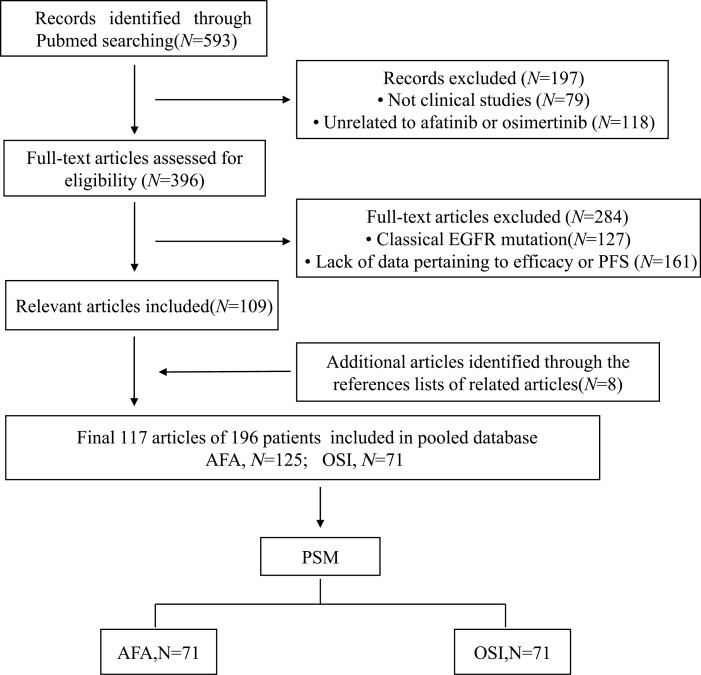
The flow chart of the study process. AFA, afatinib; BM, brain metastases; OSI, Osimertinib; PSM, propensity score matching.

A total of 593 potentially relevant articles were identified from the PubMed database. After screening the titles/abstracts and full text, 117 articles were eventually included in this study (Supplementary Table S1). The screening procedure is depicted in Fig. 1.

### Patient and EGFR Mutations

There are 196 patients were included in the pooled analysis. Chi-square analysis of the baseline characteristics between the 2 groups revealed a significant imbalance in ethnicity, pretreatment brain metastasis status, and the number of mutations (Supplementary Table S2). Subsequently, applying PSM, 71 patient pairs with similar baseline characteristics were matched (Table 1). There were more women than men (females 84, 59.2%; males 58, 40.8%), and most of them were Asian (125, 88.0%). Half of the patients were free of brain metastases before AFA/OSI treatment. In terms of mutation types, there were 73 patients (51.4%) with compound EGFR mutations, similar to that of a single one (69, 48.6%). A total of 83 (58.5%) patients received second or later-line AFA/OSI treatment, which was slightly more than the number of patients who received first-line therapy. Among these 142 patients, a total of 76 distinct types of EGFR mutations were found. The top 6 EGFR mutation types were ex20ins (23, 16.2%), 19del (9,6.3%), 19del/G724S (6, 4.2%), 18del (5, 3.5%), G719A (5, 3.5%), L861Q (5, 3.5%), G719S (4, 2.8%), S768I (4, 2.8%), L747P (3, 2.1%), L858R/T790L (3, 2.1%), and L858R/T854A (3, 2.1%) (Supplementary Fig. S1).

**Table 1.** Baseline characteristics of patients after PSM.

Characteristics	Total (*N* = 142)	AFA (*N* = 71)	OSI (*N* = 71)	*P*
Age				
Median, years (range)	59 (25-84)	58 (34-84)	61 (25-83)	
<65	89 (62.7%)	45	44	.862
≥65	53 (37.3%)	26	27	
Gender				
Male	58 (40.8)	30	28	.733
Female	84 (59.2)	41	43	
Ethnicity				
Asian	125 (88.0%)	60	65	.196
Non-Asian	17 (12.0%)	11	6	
Smoking				
Yes	43 (30.3%)	20	23	.853
No	83 (58.5%)	43	40	
N/A	16 (11.3%)	8	8	
Stage				
III	7 (4.95)	3	4	.698
Ⅳ	135 (95.1%)	68	67	
BM before AFA/OSI				
Yes	54 (38.0%)	23	31	.249
No	71 (50.0%)	37	34	
N/A	17 (12.0%)	11	6	
Mutation types				
Single	69 (48.6%)	37	32	.401
Compound	73 (51.4%)	34	39	
AFA/OSI line				
1 L	59 (41.5%)	32	27	.395
≥ 2 L	83 (58.5%)	39	44	

Abbreviations: AFA, afatinib; BM, brain metastases; N/A, not available; OSI, osimertinib; PSM, propensity score matching.

### Clinical Outcomes

**Figure 2.**
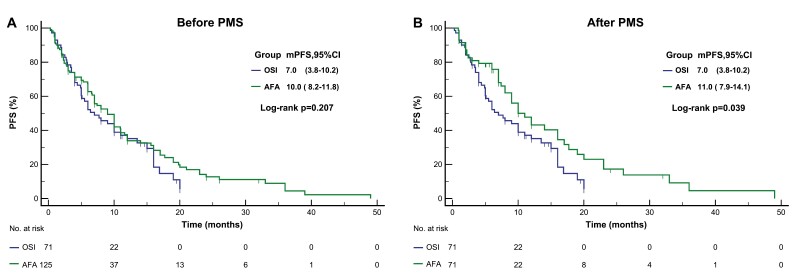
Kaplan-Meier curves for progression free survival (PFS) of comparing afatinib and osimertinib before (**A**), and after (**B**) propensity score matching. AFA, afatinib; OSI, Osimertinib; PSM, propensity score matching.

There were 125 patients in the afatinib cohort and 71 in the osmertinib cohorts were included for the analysis of efficacy and survival. Overall, the afatinib group had an ORR of 52.8%, which was marginally greater than that of the osimertinib group (43.7%). Nevertheless, there was no statistical difference (*P* = .633, Table 2). In terms of survival, the afatinib group yielded a median PFS (mPFS) of 10.0 months (95% CI: 8.2-11.8 months), which was superior in comparison to the osimertinib group, which was 7.0 months (95%CI: 3.8-10.2 months). Regrettably, no statistical difference was found (*P* = .207) (Fig. 2A). After PSM, 43 patients in the afatinib group obtained Ora of tumor response, with an ORR of 60.6%, and 40 cases in the osimertinib group obtained Ora, with an ORR of 56.3%. There was no statistically significant difference between the 2 cohorts (*P* = .609, Table 2). As for PFS, patients treated with afatinib showed a significant PFS benefit, with an mPFS of 11.0 months (95% CI: 7.9-14.1 months), superior to 7.0 months in whom received osimertinib (95% CI: 3.8-10.2 months*, P *= .039, Fig. 2B). Considering that the treatment response was heterogeneous to 1st and 2nd generation EGFR-TKI in patients with 20ins that some subtypes within the α-C-helix are sensitive to approved EGFR-TKI, while loop insertion mutations within the loop region are less responsive to treatment. And that the FDA has authorized the administration of amivantamab and Mobocertinib in the treatment of patients with NSCLC with 20ins. We compared the efficacy of AFA and OSI in a non-20ins population. After PSM, a total of 55 pairs of patients were included in the analysis. Consistent with the results in the whole population. There was no statistically significant difference between the 2 cohorts (Ora:1.26, 95%CI: 0.54-2.48, *P* = .699). However, patients treated with afatinib showed a significant PFS benefit (HR:0.60, 95%CI: 0.37-0.99, *P* = .047), especially in Asian populations and in patients carrying multiple mutations (Supplementary Fig. S2). In addition, we evaluated the clinical benefit in terms of ORR and PFS according to some important factor that may affect the overall outcome. For patients receiving first-line treatment, the ORR and mPFS were 70.0% and 15.0 months, respectively, which were significantly better than those of patients receiving second-line and later treatments, which were 43.1% and 6.0 months, respectively. Patients carrying a single mutation had significantly superior ORR than those carrying compound mutations (59.8% vs. 45.6%, *P* = .049). However, there was no statistical difference in prognosis, although patients with a single mutation showed a longer mPFS (10.0 months vs. 7.7 months, *P* = .116). For patients with non-20ins mutations, their mPFS was significantly longer than that of patients with 20ins (10.0 months vs. 5.1 months, *P* = .034), although there was no statistical difference regarding ORR (54.8% vs. 51.2%, *P* = .679) (Supplementary Table S3).

**Table 2.** Efficacy and PFS before and after PSM.

Characteristics	Before PSM (*N* = 196)			After PSM (*N* = 142)		
	AFA (*N* = 125)	OSI (*N* = 71)	*P*	AFA (*N* = 71)	OSI (*N* = 71)	*P*
Efficacy						
CR	1 (0.8%)	2 (2.8%)	—	1 (1.4%)	2 (2.8%)	—
PR	63 (50.4%)	38 (53.5%)	—	42 (59.2%)	38 (53.5%)	—
SD	37 (29.6%)	19 (26.8%)	—	18 (25.4%)	19 (26.8%)	—
PD	22 (17.6%)	12 (16.9%)	—	10 (14.1)	12 (16.9%)	—
OR	66 (52.8%)	40 (43.7%)	.633	43 (60.6%)	40 (56.3%)	.609
PFS						
Median (months)	10.0	7.0	.207	11.0	7.0	.039
95% CI	8.2-11.8	3.8-10.2	—	7.9-14.1	3.8-10.2	—

Abbreviations: AFA, afatinib; OSI, Osimertinib; PSM, propensity score matching.

### Subgroup Analysis

**Figure 3.**
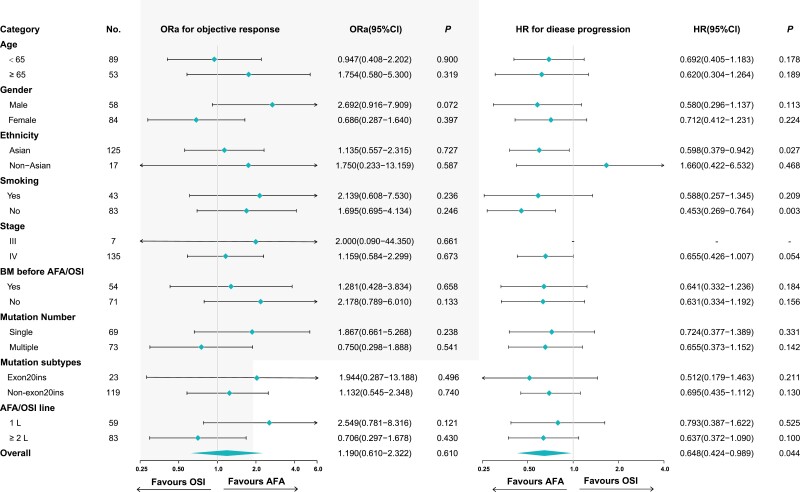
Comparison of treatment response and progression-free survival (PFS) between afatinib versus osimertinib in multiple subgroups (osimertinib group set as the reference group). AFA, afatinib; BM, brain metastases; OSI, osimertinib.

Subgroup analysis was conducted for efficacy and survival to further investigate whether there were prospective patients more probable to benefit from the treatment of AFA or OSI. However, in terms of age, sex, ethnicity, smoking history, tumor stage, brain metastases, mutation number, and treatment line of AFA/OSI, there was no significant difference in the efficacy of afatinib versus osimertinib (Fig. 3). Subgroup analysis of survival showed that among Asian patients, afatinib treatment was correlated with a greater PFS than osimertinib (HR: 0.598, 95% CI: 0.379-0.947, *P* = .027). Likewise, in non-smoking patients, afatinib treatment was correlated with a superior prognosis than osimertinib (HR: 0.453, 95% CI: 0.269-0.764, *P* = .003, Fig. 3).

**Figure 4.**
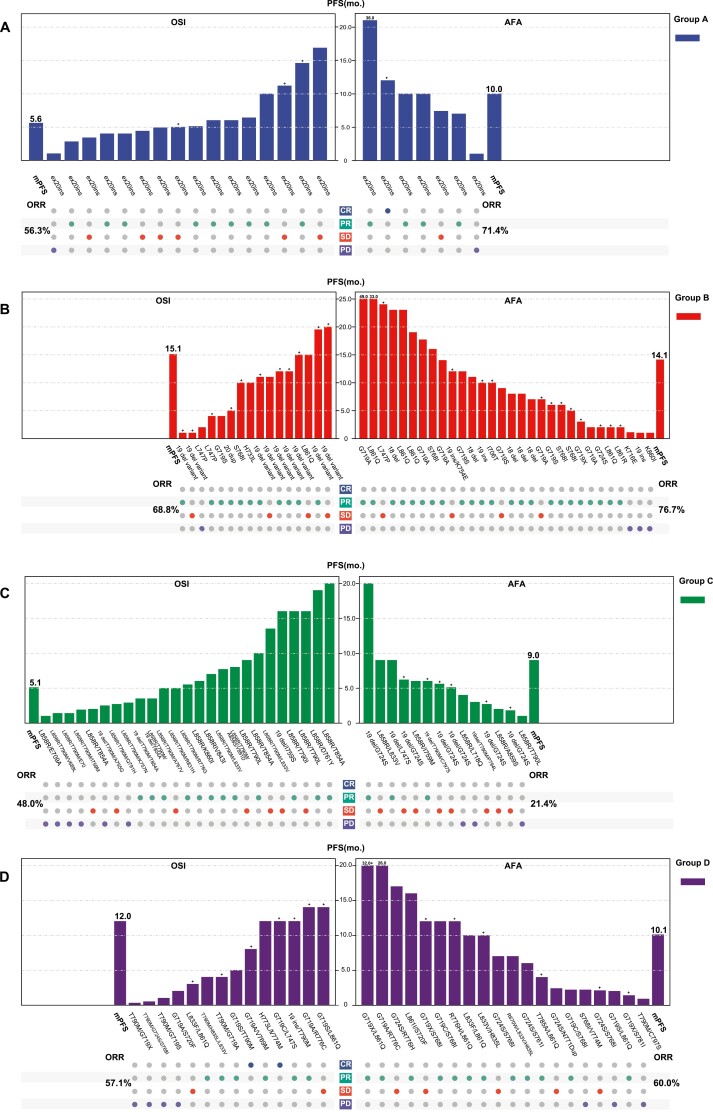
Tumor response and progression-free survival (PFS) of each patient as well as overall tumor objective response rate (ORR) and median PFS for each subgroup: group A, exon 20 insertions; group B, other single uncommon EGFR mutations; group C, compound EGFR mutations that with 19del/L858; group D, compound EGFR mutations that without 19del/L858R (left for osimertinib group, right for afatinib group).

**Figure 5.**
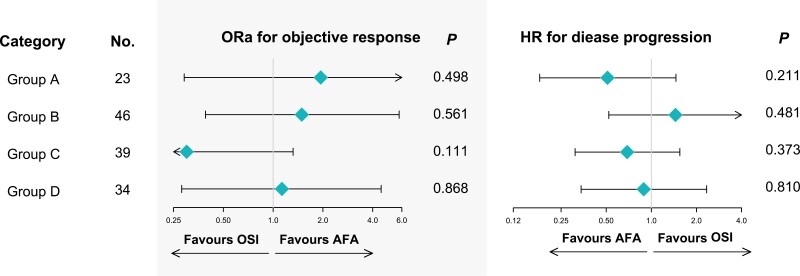
Comparison of treatment response and progression-free survival (PFS) between afatinib versus osimertinib according to mutation patterns (osimertinib group set as the reference group).

Next, explorative subgroup analysis was conducted in patients with different mutation types: group A for ex20ins, group B for non-ex20ins single uncommon EGFR mutations, group C for compound EGFR mutations containing 19del/L858R, and group D for compound EGFR mutations without 19del/L858R. The baseline characteristics of each group are present in Supplementary Table S4. The tumor responses to AFA/OSI and PFS of each patient in different groups are shown in Fig. 4. Patients in group B exhibited the best efficacy and prognosis, treated with either afatinib or osimertinib. However, patients in group C presented the worst ORR and PFS. Overall, the ORR and mPFS for patients with afatinib treatment were 71.4% and 10.0 months, 76.7% and 14.1 months, 21.4% and 9.0 months, and 60.0% and 10.1 months, in group A-D, respectively. The corresponding values for those patients treated with osimertinib were 56.3% and 5.6 months, 68.8% and 15.1 months, 48.0% and 5.1 months, and 57.1% and 12.0 months, respectively (Fig. 4). The results of the univariate logistic regression model for ORR as well as Cox proportional hazards model for PFS showed that neither afatinib nor osimertinib displayed an efficacy advantage or survival benefit over each other in each subgroup of uncommon EGFR mutations patterns (all *P* < .05, Fig. 5).

## Discussion

Our analysis originally compared the outcomes of afatinib and osimertinib treatment in patients with NSCLC harboring uncommon EGFR mutations. The present analyses revealed an ORR of 60.6% in patients receiving afatinib, which was similar to that in patients who received osimertinib (ORR: 56.3%). However, the afatinib group showed a significant PFS benefit, with an mPFS of 11.0 months, superior to that of the osimertinib group (7.0 months). In addition, neither afatinib nor osimertinib expressed a remarkable effectiveness benefit or PFS advantage over each other regardless of the mutation types.

Afatinib and osimertinib are active in the treatment of patients with NSCLC who carry uncommon EGFR mutations, according to the observations of clinical trials and research conducted in the real world.^8,9,12,13^ However, given the low mutation frequency as well as high heterogeneity of uncommon EGFR mutations, there have been no clinical studies comparing the efficacies of afatinib and osimertinib in patients with NSCLC harboring uncommon EGFR mutations. In relation to this theme, preclinical studies have shown that, compared to osimertinib, afatinib exhibited relatively broad activity against uncommon EGFR mutations.^14,15^ Consistently, in the present analysis, we observed a better PFS benefit in patients who received afatinib than that in those receiving osimertinib. However, the efficacy was not significantly different between them, although there was a slight advantage in the afatinib cohort. Our findings were in line with those of a previous study that compared the effectiveness of afatinib and osimertinib in treating patients with NSCLC who had classical EGFR mutations.^16^ This study investigated 326 osimertinib-treated patients and 224 afatinib-treated patients. The afatinib cohort had a significant overall survival (OS) benefit with an mOS of 36.2 months (95% CI: 30.6-55.3 months) compared to the osimertinib cohort (25.1 months, 95% CI not estimated). Presently, there are insufficient clinical data to compare the effectiveness of afatinib and osimertinib. Nevertheless, afatinib is more efficacious than osimertinib in patients with uncommon EGFR mutations, according to the current clinical data from real-world retrospective studies as well as case series reports ^17,18^

Previous research have indicated that the pattern of mutations and the presence of partner mutated genes are closely associated with the EGFR-TKIs’ inconsistent efficacy against uncommon EGFR mutations.^4-7^ In the post hoc investigation of the LUX-lung clinical studies, 23 patients with ex20ins and afatinib treatment had an ORR of 8.7% and a PFS of only 2.7 months. In contrast, the ORRs for patients with G719X, S768I, and L861Q were 78%, 100%, and 56%, respectively, with the corresponding mPFS of 13.8, 14.7, and 8.2 months, respectively, representing the best efficacy of afatinib.^8^ A Spanish multicenter retrospective study also showed that afatinib had significantly lower efficacy in patients carrying ex20ins than that in patients with other mutation types, yielding an ORR of 13.0% with an mOS at 10.7 months.^19^ For osimertinib, ORR was reported to be 5.0%, and the mPFS was 3.6 months in real-world retrospective research that included 21 patients harboring ex20ins.^20^ Patients with the G719X, S768I, and L861Q mutations in the KCSG-LU15-09 trial experienced ORRs of 53.0%, 78.0%, and 38.0% after receiving osimertinib, with correlating mPFS of 8.2, 15.2, and 12.3 months, respectively.^9^ An international case series study (UNICORN) including 60 patients reported that the response rate and PFS were 78%, at 15.7 months and 53%, 8.6 months, respectively, in patients with L861Q and G719X.^10^ Another study with a small sample showed that the ORR of patients with compound EGFR mutations that contained G719X was 62.5% with an mPFS of 13.7 months.^11^

Tumor cells may harbor not only a single mutation in EGFR but also a combination of mutations known as compound EGFR mutations.^7,21^ The ORR for patients with compound EGFR mutations who received afatinib was 77.1%, with a median time to treatment failure at 14.7 months, according to a pooled analysis of 315 patients with NSCLC.^12^ Similarly, the Spanish multicenter retrospective study also reported a favorable ORR of 70.0% with an mOS of 28.8 months in the afatinib-treated patients carrying compound EGFR mutations.^19^ For osimertinib, a retrospective study reported a much inferior tumor response and a shorter mPFS that patients harboring compound EGFR mutations yield an ORR of 27% with an mPFS of 5.0 months following osimertinib.^22^ The UNICORN study reported an ORR of 61%, mPFS of 9.5 months, and median overall survival of 24.5 months in patients who received OSI as first-line treatment.^10^ Another study reported that the ORR for first-line OSI treatment was 63.6% with an mPFS of 5.5 months.^11^ The clinical findings discussed above were supported by preclinical findings that compound EGFR mutations seem to have been fairly more insensitive to afatinib than osimertinib.^23^ In addition, Previous research found that patients whose compound EGFR mutations contained 19del/L858R had a better ORR and prognosis than those who do not.^7,12,21^

In this analysis, we performed subgroup analyses to evaluate the treatment efficiency and prognosis in patients with different uncommon EGFR mutation types to determine which, if any, prospective subgroups of the population were most likely to benefit from afatinib or osimertinib treatment. However, disappointingly, no superior efficacy advantage or PFS benefit was observed. We did, nevertheless, found that patients with single, non-ex20ins, uncommon EGFR mutations had the best outcome, with either afatinib or osimertinib treatment, which was consistent with previous findings. However, contrary to the findings of previous studies, we found that patients harboring compound EGFR mutations without 19del/L858R had a superior tumor response and a lengthier PFS timing than those with 19del/L858R. We could not entirely exclude that the benefit in this cohort of patients was driven by the accompanied major uncommon EGFR mutations, which were demonstrated to be sensitive to afatinib and osimertinib.

Evidence from clinical trials demonstrated that both afatinib and osimertinib showed favorable intracranial efficacy against brain metastases in patients with NSCLC who harbor classical EGFR mutations and could improve central nervous system remission rates.^24-27^ However, preclinical studies indicated that, compared to afatinib, osimertinib showed a higher blood-brain barrier penetration capability.^28^ Osimertinib was discovered to provide a greater clinical benefit than afatinib in patients with brain metastases, according to clinical studies conducted in patients with NSCLC who had classical EGFR mutations.^16^ Presently, available data that concentrates on the clinical efficacy of afatinib and osimertinib against brain metastases in patients carrying uncommon EGFR mutations are limited. The UNICORN study reported a brain ORR of 46% in 13 patients with an evaluable response of brain metastases.^10^Inger et al^11^ reported an intracranial ORR of 36.4% with an intracranial PFS at 6.1 months in 11 patients. In the current analysis, we carried out a subgroup analysis to examine the efficacy of afatinib and osimertinib in patients harboring uncommon EGFR mutations and different brain metastasis statuses. Inconsistent with the results of preclinical studies and studies in classical EGFR mutation populations, afatinib did not display a superior efficacy advantage over osimertinib in patients with or without brain metastases.

There were some unavoidable limitations of this analysis. This was publication-based reanalysis research. This may be impacted by a variety of uncontainable confounding variables, including but not limited to selection bias, publication bias, and others. Second, with only 17 non-Asian patients, the applicability of the results of this analysis to Caucasian populations remains limited. Third, because of the diverse nature of the articles that are included, some potential clinical factors that may affect efficacy, such as performance status, co-mutations in genes other than EGFR, history of prior TKI therapy, and the dose of AFA/OSI were not entirely available, which may affect the stability of the results. In addition, afatinib was introduced into the clinical years before osimertinib, which may result in a potential bias for reports of long-term responders in favor of afatinib. Then, the detection rate of EGFR mutations and the reliability of results may be impacted by the lack of clarity surrounding sample sources, testing platforms, and techniques, such as RT-PCR and NGS, for EGFR testing.

## Conclusion

Both afatinib and osimertinib treatments exhibit favorable tumor responses and PFS benefits in patients with NSCLC who carry uncommon EGFR mutations. Afatinib showed a more profound and durable PFS benefit than osimertinib, although no efficacy advantage was manifested.

## Supplementary Material

oyad111_suppl_Supplementary_Figure_1

oyad111_suppl_Supplementary_Figure_2

oyad111_suppl_Supplementary_Tables

oyad111_suppl_Supplementary_Figure_Captions

## Data Availability

The data underlying this article will be shared on reasonable request to the corresponding author.
