# Supplementary material for: Clinical Outcomes of Afatinib Versus Osimertinib in Patients With Non-Small Cell Lung Cancer With Uncommon EGFR Mutations: A Pooled Analysis
Source: Oncologist. 2023 Apr 28;28(6):e397–405. doi: 10.1093/oncolo/oyad111 (PMC10243768; doi:10.1093/oncolo/oyad111)
Supplement: oyad111_suppl_Supplementary_Figure_1 [file oyad111_suppl_supplementary_figure_1.pdf]

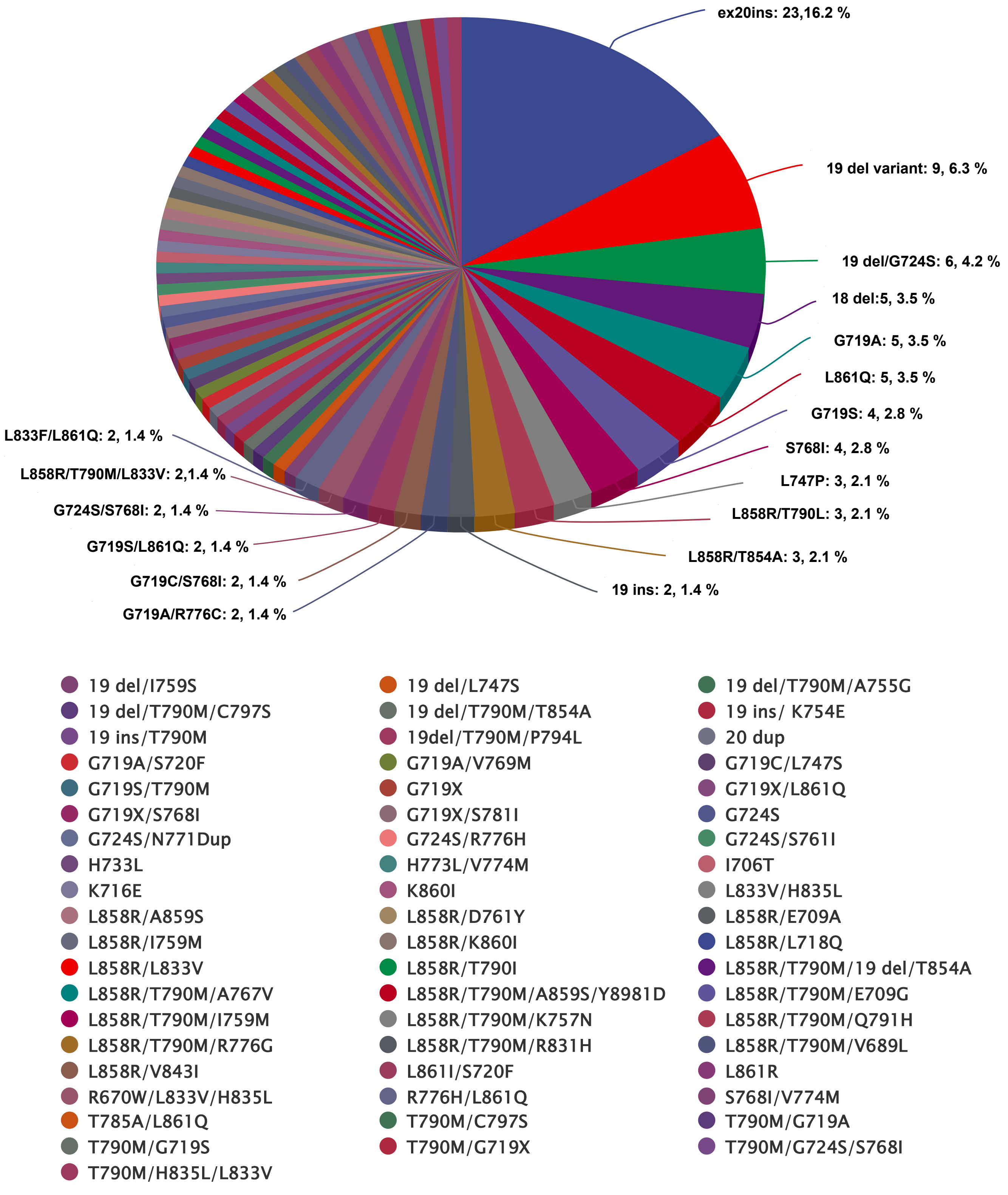
ex20ins: 23,16.2 %
19 del variant: 9, 6.3 %
19 del/G724S: 6, 4.2 %
18 del:5, 3.5 %
G719A: 5, 3.5 %
L861Q: 5, 3.5 %
G719S: 4, 2.8 %
S768I: 4, 2.8 %
L747P: 3, 2.1 %
L858R/T790L: 3, 2.1 %
L858R/T854A: 3, 2.1 %
19 ins: 2, 1.4 %
L833F/L861Q: 2, 1.4 %
L858R/T790M/L833V: 2,1.4 %
G724S/S768I: 2, 1.4 %
G719S/L861Q: 2, 1.4 %
G719C/S768I: 2, 1.4 %
G719A/R776C: 2, 1.4 %
19 del/I759S
19 del/L747S
19 del/T790M/A755G
19 del/T790M/C797S
19 del/T790M/T854A
19 ins/ K754E
19 ins/T790M
19del/T790M/P794L
20 dup
G719A/S720F
G719A/V769M
G719C/L747S
G719S/T790M
G719X
G719X/L861Q
G719X/S768I
G719X/S781I
G724S
G724S/N771Dup
G724S/R776H
G724S/S761I
H733L
H773L/V774M
I706T
K716E
K860I
L833V/H835L
L858R/A859S
L858R/D761Y
L858R/E709A
L858R/I759M
L858R/K860I
L858R/L718Q
L858R/L833V
L858R/T790I
L858R/T790M/19 del/T854A
L858R/T790M/A767V
L858R/T790M/A859S/Y8981D
L858R/T790M/E709G
L858R/T790M/I759M
L858R/T790M/K757N
L858R/T790M/Q791H
L858R/T790M/R776G
L858R/T790M/R831H
L858R/T790M/V689L
L858R/V843I
L861I/S720F
L861R
R670W/L833V/H835L
R776H/L861Q
S768I/V774M
T785A/L861Q
T790M/C797S
T790M/G719A
T790M/G719S
T790M/G719X
T790M/G724S/S768I
T790M/H835L/L833V
