# Supplementary figures and images for: Clinical Outcomes of Afatinib Versus Osimertinib in Patients With Non-Small Cell Lung Cancer With Uncommon EGFR Mutations: A Pooled Analysis
Source: Oncologist. 2023 Apr 28;28(6):e397–405. doi: 10.1093/oncolo/oyad111 (PMC10243768; doi:10.1093/oncolo/oyad111)

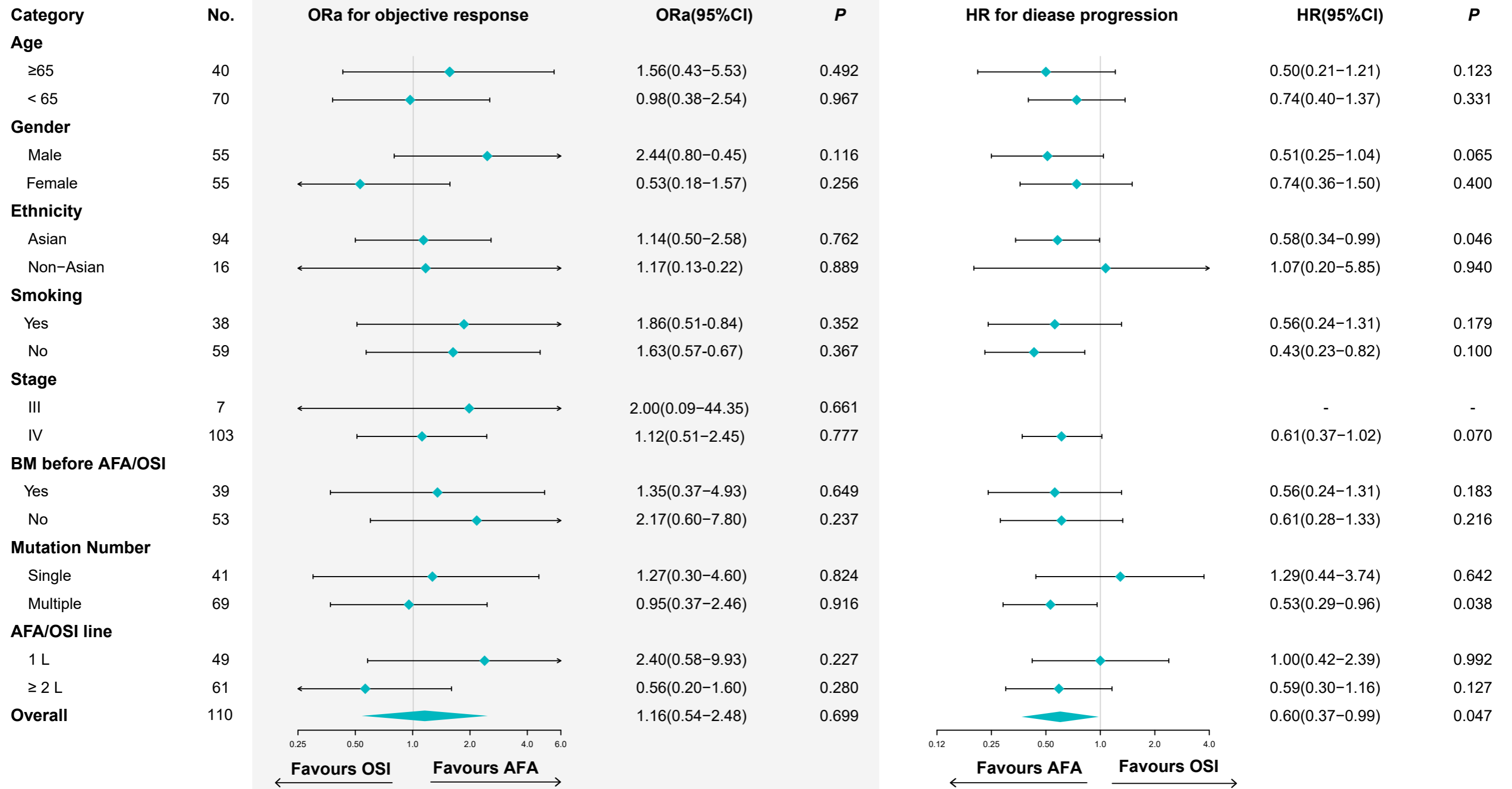

Supplement: oyad111_suppl_Supplementary_Figure_2 [file oyad111_suppl_supplementary_figure_2.pdf]
