# Supplementary material for: Clinical Outcomes of Afatinib Versus Osimertinib in Patients With Non-Small Cell Lung Cancer With Uncommon EGFR Mutations: A Pooled Analysis
Source: Oncologist. 2023 Apr 28;28(6):e397–405. doi: 10.1093/oncolo/oyad111 (PMC10243768; doi:10.1093/oncolo/oyad111)
Supplement: oyad111_suppl_Supplementary_Tables [file oyad111_suppl_supplementary_tables.docx]

Supplementary Table 1. The studies included for this pooled analysis

| Study | Study type | years | No. of patients | EGFR-TKIs | References* |
| --- | --- | --- | --- | --- | --- |
| Chinese retrospective study | Single-Center China study in patients with uncommon EGFR L747P and L747S mutations | 2019 | 3 | afatinib | ^1^ |
| Germany retrospective study | Single-Center Germany study in patients with uncommon EGFR mutations treated with afatinib | 2019 | 2 | afatinib | ^2^ |
| Korea retrospective study | Single-Center Korea study in patients with EGFR Exon 20 Insertion Mutations treated with EGFR TKIs | 2015 | 2 | afatinib | ^3^ |
| Germany retrospective study | Single-Center Germany study in patients with rare or complex EGFR mutations treated with EGFR TKIs | 2019 | 3 | afatinib | ^4^ |
| Chinese retrospective study | Single-Center China study in patients with uncommon EGFR mutations treated with afatinib | 2018 | 5 | afatinib | ^5^ |
| Japanese retrospective study | Single-Center Japan study in patients with complex EGFR mutations treated with afatinib | 2017 | 1 | afatinib | ^6^ |
| Chinese retrospective study | Single-Center China study in patients with uncommon EGFR mutations treated with afatinib | 2020 | 3 | afatinib | ^7^ |
| Japanese retrospective study | Single-Center Japan study in patients with uncommon EGFR mutations | 2019 | 9 | afatinib | ^8^ |
| India retrospective study | Single-Center study in patients with rare EGFR mutations | 2021 | 4 | afatinib | ^9^ |
| Chinese retrospective study | Single-Center China study in patients with uncommon EGFR mutations treated with afatinib | 2020 | 2 | afatinib | ^10^ |
| Chinese retrospective study | Single-Center China study in patients with uncommon EGFR mutations treated with afatinib | 2018 | 2 | afatinib | ^11^ |
| Italy retrospective study | Single-Center study in patients with uncommon EGFR mutations | 2018 | 1 | afatinib | ^12^ |
| Chinese retrospective study | Multiple-Center China study in patients with EGFR Exon 20 Insertion Mutations treated with Afatinib | 2021 | 8 | afatinib | ^13^ |
| Case reports/case series | Various(n=73) | 2015-2022 | 76 | afatinib | ^14-86^ |
| Chinese retrospective study | Single-Center China study in patients with uncommon EGFR mutations treated with afatinib | 2020 | 11 | afatinib | ^10^ |
| Chinese retrospective study | Single-Center China study in patients with exon 20 inertion treated with osimertinib | 2019 | 6 | osimertinib | ^87^ |
| Chinese retrospective study | Single-Center China study in patients with exon 19 insertion-deletion variants | 2020 | 8 | osimertinib | ^88^ |
| Case reports/case series | Various(n=73) | 2018-2022 | 46 | osimertinib | ^3, 9, 26, 31, 36, 55, 58, 63, 69, 79, 84, 86, 89-117^ |

*: References here refer to the articles included in this pooled-analysis, not the references cited in the manuscript, which are listed in the reference section of the manuscript.

**References**

1. Liang SK, Ko JC, Yang JC, et al. Afatinib is effective in the treatment of lung adenocarcinoma with uncommon EGFR p.L747P and p.L747S mutations. *Lung Cancer* 2019; 133: 103-109. 2019/06/16. DOI: 10.1016/j.lungcan.2019.05.019.

2. Martin J, Lehmann A, Klauschen F, et al. Clinical Impact of Rare and Compound Mutations of Epidermal Growth Factor Receptor in Patients With Non-Small-Cell Lung Cancer. *Clin Lung Cancer* 2019; 20: 350-362 e354. 2019/06/09. DOI: 10.1016/j.cllc.2019.04.012.

3. Byeon S, Kim Y, Lim SW, et al. Clinical Outcomes of EGFR Exon 20 Insertion Mutations in Advanced Non-small Cell Lung Cancer in Korea. *Cancer Res Treat* 2019; 51: 623-631. 2018/07/28. DOI: 10.4143/crt.2018.151.

4. Kauffmann-Guerrero D, Huber RM, Reu S, et al. NSCLC Patients Harbouring Rare or Complex EGFR Mutations Are More Often Smokers and Might Not Benefit from First-Line Tyrosine Kinase Inhibitor Therapy. *Respiration* 2018; 95: 169-176. 2017/12/01. DOI: 10.1159/000484175.

5. Wang DD, Lee VH, Zhu G, et al. Selectivity profile of afatinib for EGFR-mutated non-small-cell lung cancer. *Mol Biosyst* 2016; 12: 1552-1563. 2016/03/11. DOI: 10.1039/c6mb00038j.

6. Ishii H, Azuma K, Sakai K, et al. Determination of Somatic Mutations and Tumor Mutation Burden in Plasma by CAPP-Seq during Afatinib Treatment in NSCLC Patients Resistance to Osimertinib. *Sci Rep* 2020; 10: 691. 2020/01/22. DOI: 10.1038/s41598-020-57624-4.

7. Ma C, Zhang J, Tang D, et al. Tyrosine Kinase Inhibitors Could Be Effective Against Non-small Cell Lung Cancer Brain Metastases Harboring Uncommon EGFR Mutations. *Front Oncol* 2020; 10: 224. 2020/03/21. DOI: 10.3389/fonc.2020.00224.

8. Ikemura S, Yasuda H, Matsumoto S, et al. Molecular dynamics simulation-guided drug sensitivity prediction for lung cancer with rare EGFR mutations. *Proc Natl Acad Sci U S A* 2019; 116: 10025-10030. 2019/05/03. DOI: 10.1073/pnas.1819430116.

9. Mehta A and Vasudevan S. Rare epidermal growth factor receptor gene alterations in non-small cell lung cancer patients, tyrosine kinase inhibitor response and outcome analysis. *Cancer Treat Res Commun* 2021; 28: 100398. 2021/05/31. DOI: 10.1016/j.ctarc.2021.100398.

10. Lin YT, Tsai TH, Wu SG, et al. Complex EGFR mutations with secondary T790M mutation confer shorter osimertinib progression-free survival and overall survival in advanced non-small cell lung cancer. *Lung Cancer* 2020; 145: 1-9. 2020/05/11. DOI: 10.1016/j.lungcan.2020.04.022.

11. Tsai MJ, Hung JY, Lee MH, et al. Better Progression-Free Survival in Elderly Patients with Stage IV Lung Adenocarcinoma Harboring Uncommon Epidermal Growth Factor Receptor Mutations Treated with the First-line Tyrosine Kinase Inhibitors. *Cancers (Basel)* 2018; 10 2018/11/16. DOI: 10.3390/cancers10110434.

12. Improta G, Zupa A, Natalicchio MI, et al. Uncommon frame-shift exon 19 EGFR mutations are sensitive to EGFR tyrosine kinase inhibitors in non-small cell lung carcinoma. *Med Oncol* 2018; 35: 28. 2018/02/02. DOI: 10.1007/s12032-018-1078-7.

13. Wei Y, Jiang B, Liu S, et al. Afatinib as a Potential Therapeutic Option for Patients With NSCLC With EGFR G724S. *JTO Clin Res Rep* 2021; 2: 100193. 2021/10/01. DOI: 10.1016/j.jtocrr.2021.100193.

14. Iwamoto Y, Ichihara E, Hara N, et al. Efficacy of afatinib treatment for lung adenocarcinoma harboring exon 18 delE709_T710insD mutation. *Jpn J Clin Oncol* 2019; 49: 786-788. 2019/06/13. DOI: 10.1093/jjco/hyz086.

15. Zeng L, Zhang Y and Yang N. EGFR exon 18 DelE709_T710insD as an Acquired Resistance Mechanism to Afatinib in an Advanced EGFR exon 18 E709H Lung Adenocarcinoma. *J Thorac Oncol* 2018; 13: e93-e95. 2018/05/26. DOI: 10.1016/j.jtho.2018.01.006.

16. Zhou T, Zhou X, Li P, et al. EGFR L747P mutation in one lung adenocarcinoma patient responded to afatinib treatment: a case report. *J Thorac Dis* 2018; 10: E802-E805. 2019/02/13. DOI: 10.21037/jtd.2018.12.26.

17. Agatsuma N, Yasuda Y and Ozasa H. Malignant Pleural Mesothelioma Harboring Both G719C and S768I Mutations of EGFR Successfully Treated with Afatinib. *J Thorac Oncol* 2017; 12: e141-e143. 2017/08/26. DOI: 10.1016/j.jtho.2017.04.028.

18. Ma C, Huang C, Tang D, et al. Afatinib for Advanced Non-small Cell Lung Cancer in a Case With an Uncommon Epidermal Growth Factor Receptor Mutation (G719A) Identified in the Cerebrospinal Fluid. *Front Oncol* 2019; 9: 628. 2019/08/10. DOI: 10.3389/fonc.2019.00628.

19. Niogret J, Coudert B and Boidot R. Primary Resistance to Afatinib in a Patient with Lung Adenocarcinoma Harboring Uncommon EGFR Mutations: S768I and V769L. *J Thorac Oncol* 2018; 13: e113. 2018/06/25. DOI: 10.1016/j.jtho.2018.02.004.

20. Russo A, Franchina T, Ricciardi GRR, et al. Rapid Acquisition of T790M Mutation after Treatment with Afatinib in an NSCLC Patient Harboring EGFR Exon 20 S768I Mutation. *J Thorac Oncol* 2017; 12: e6-e8. 2016/12/19. DOI: 10.1016/j.jtho.2016.09.132.

21. Chan RT. Afatinib for an EGFR exon 20 insertion mutation: A case report of progressive stage IV metastatic lung adenocarcinoma with 54 months' survival. *Asia Pac J Clin Oncol* 2018; 14 Suppl 1: 7-9. 2018/03/07. DOI: 10.1111/ajco.12853.

22. Zhu X, Bai Q, Lu Y, et al. Response to Tyrosine Kinase Inhibitors in Lung Adenocarcinoma with the Rare Epidermal Growth Factor Receptor Mutation S768I a Retrospective Analysis and Literature Review. *Target Oncol* 2017; 12: 81-88. DOI: 10.1007/s11523-016-0455-4.

23. Zhang H, Shao YW and Xia Y. Responsiveness to Full-Dose Afatinib in a Patient With Lung Adenocarcinoma Harboring EGFR S768I and V769L Mutations. *J Thorac Oncol* 2019; 14: e25-e27. 2019/01/27. DOI: 10.1016/j.jtho.2018.10.165.

24. Coupkova H and Vyzula R. Afatinib in the Treatment of Advanced Non-Small Cell Lung Cancer with Rare EGFR (in exon 18-T179X) Mutation - a Case Report. *Klin Onkol* 2018; 31: 380-383. 2018/12/14. DOI: 10.14735/amko2018380.

25. Nakamura D, Miura K, Kumeda H, et al. Successful Resection of G719X-Positive Pleomorphic Carcinoma after Afatinib Treatment. *Case Rep Oncol* 2017; 10: 1035-1040. 2018/03/09. DOI: 10.1159/000480120.

26. Cai Y, Wang X, Guo Y, et al. Successful treatment of a lung adenocarcinoma patient with a novel EGFR exon 20-ins mutation with afatinib: A case report. *Medicine (Baltimore)* 2019; 98: e13890. 2019/01/05. DOI: 10.1097/MD.0000000000013890.

27. Taniguchi Y, Yamamoto M, Ikushima H, et al. Successful Treatment of Afatinib-Refractory Non-Small Cell Lung Cancer with Uncommon Complex EGFR Mutations Using Pembrolizumab: A Case Report. *Case Rep Oncol* 2019; 12: 564-567. 2019/08/21. DOI: 10.1159/000501848.

28. Iida Y, Kumasawa F, Shimizu T, et al. Successful treatment of an elderly patient with an uncommon L861Q epidermal growth factor receptor mutation with low-dose afatinib: A case report. *Thorac Cancer* 2020; 11: 447-450. 2019/11/30. DOI: 10.1111/1759-7714.13269.

29. Ikeuchi T, Tokuyasu H and Ishikawa S. Successful Treatment of Lung Adenocarcinoma with Epidermal Growth Factor Receptor Compound Mutations Involving Exon 19 Deletion and Exon 20 Insertion by Afatinib. *Intern Med* 2019; 58: 101-104. 2019/01/05. DOI: 10.2169/internalmedicine.0927-18.

30. Furuya T, Shimada J, Okada S, et al. Successful treatment with afatinib for pancreatic metastasis of lung adenocarcinoma: a case report. *J Thorac Dis* 2017; 9: E890-E893. 2017/12/23. DOI: 10.21037/jtd.2017.09.123.

31. Qin BD, Jiao XD, Yuan LY, et al. The effectiveness of afatinib and osimertinib in a Chinese patient with advanced lung adenocarcinoma harboring a rare triple EGFR mutation (R670W/H835L/L833V): a case report and literature review. *Onco Targets Ther* 2018; 11: 4739-4745. 2018/08/22. DOI: 10.2147/OTT.S167346.

32. Watanabe M, Oizumi S, Kiuchi S, et al. The Effectiveness of Afatinib in a Patient with Advanced Lung Adenocarcinoma Harboring Rare G719X and S768I Mutations. *Intern Med* 2018; 57: 993-996. 2017/12/12. DOI: 10.2169/internalmedicine.9565-17.

33. Galli G, Corrao G, Imbimbo M, et al. Uncommon mutations in epidermal growth factor receptor and response to first and second generation tyrosine kinase inhibitors: A case series and literature review. *Lung Cancer* 2018; 115: 135-142. 2018/01/02. DOI: 10.1016/j.lungcan.2017.12.002.

34. Frega S, Lorenzi M, Fassan M, et al. Clinical features and treatment outcome of non-small cell lung cancer (NSCLC) patients with uncommon or complex epidermal growth factor receptor (EGFR) mutations. *Oncotarget* 2017; 8: 32626-32638. DOI: 10.18632/oncotarget.15945.

35. An N, Wang H, Zhu H, et al. Great efficacy of afatinib on a patient with lung adenocarcinoma harboring uncommon EGFR delE709_T710insD mutations: a case report. *Onco Targets Ther* 2019; 12: 7399-7404. 2019/11/07. DOI: 10.2147/OTT.S221638.

36. Nasu S, Shiroyama T, Morita S, et al. Osimertinib Treatment Was Unsuccessful for Lung Adenocarcinoma with G719S, S768I, and T790M Mutations. *Intern Med* 2018; 57: 3643-3645. 2018/08/28. DOI: 10.2169/internalmedicine.0923-18.

37. Zhang X, Jiang W, Yang N, et al. Afatinib response in a lung adenocarcinoma with novel compound S720F+L861R mutation in EGFR. *Lung Cancer* 2020; 148: 170-172. 2020/07/15. DOI: 10.1016/j.lungcan.2020.07.002.

38. Frega S, Conte P, Fassan M, et al. A Triple Rare E709K and L833V/H835L EGFR Mutation Responsive to an Irreversible Pan-HER Inhibitor: A Case Report of Lung Adenocarcinoma Treated with Afatinib. *J Thorac Oncol* 2016; 11: e63-e64. 2016/05/01. DOI: 10.1016/j.jtho.2016.01.023.

39. van Kempen LC, Wang H, Aguirre ML, et al. Afatinib in Osimertinib-Resistant EGFR ex19del/T790M/P794L Mutated NSCLC. *J Thorac Oncol* 2018; 13: e161-e163. 2018/04/29. DOI: 10.1016/j.jtho.2018.04.020.

40. Tamiya M, Shiroyama T, Nishihara T, et al. Afatinib successfully treated leptomeningeal metastasis during erlotinib treatment in a patient with EGFR-mutant (Exon18:G719S) lung adenocarcinoma as a second-line chemotherapy. *Asia Pac J Clin Oncol* 2017; 13: e531-e533. 2016/12/23. DOI: 10.1111/ajco.12643.

41. Tamura T, Kawakado K, Makimoto G, et al. Limited effect of afatinib in a non-small cell lung cancer patient harboring an epidermal growth factor receptor K860I missense mutation: A case report. *Thorac Cancer* 2021; 12: 1770-1774. 2021/05/05. DOI: 10.1111/1759-7714.13941.

42. Zochbauer-Muller S, Kaserer B, Prosch H, et al. Case Report: Afatinib Treatment in a Patient With NSCLC Harboring a Rare EGFR Exon 20 Mutation. *Front Oncol* 2020; 10: 593852. 2021/02/13. DOI: 10.3389/fonc.2020.593852.

43. Ibrahim U, Saqib A and Atallah JP. EGFR exon 18 delE709_T710insD mutated stage IV lung adenocarcinoma with response to afatinib. *Lung Cancer* 2017; 108: 45-47. 2017/06/20. DOI: 10.1016/j.lungcan.2017.02.023.

44. Tanizaki J, Banno E, Togashi Y, et al. Case report Durable response to afatinib in a patient with lung cancer harboring two uncommon mutations of EGFR and a KRAS mutation. *Lung Cancer* 2016; 101: 11-15. DOI: 10.1016/j.lungcan.2016.09.001.

45. Liu J, Jin B, Su H, et al. Afatinib helped overcome subsequent resistance to osimertinib in a patient with NSCLC having leptomeningeal metastasis baring acquired EGFR L718Q mutation: a case report. *BMC Cancer* 2019; 19: 702. 2019/07/19. DOI: 10.1186/s12885-019-5915-7.

46. He SY, Lin QF, Chen J, et al. Efficacy of afatinib in a patient with rare EGFR (G724S/R776H) mutations and amplification in lung adenocarcinoma: A case report. *World J Clin Cases* 2021; 9: 1329-1335. 2021/03/02. DOI: 10.12998/wjcc.v9.i6.1329.

47. Shijubou N, Sumi T, Kamada K, et al. Long-term response to afatinib in an elderly patient with uncommon epidermal growth factor receptor mutation-positive lung adenocarcinoma. *Thorac Cancer* 2021; 12: 989-992. 2021/02/04. DOI: 10.1111/1759-7714.13869.

48. Fang W, Huang Y, Gan J, et al. Durable Response of Low-Dose Afatinib plus Cetuximab in an Adenocarcinoma Patient with a Novel EGFR Exon 20 Insertion Mutation. *J Thorac Oncol* 2019; 14: e220-e221. 2019/09/29. DOI: 10.1016/j.jtho.2019.05.023.

49. Velcheti V, Khunger M and Abazeed ME. Novel EGFR Exon 18 (G721R) Mutation in a Patient with Non-Small Cell Lung Carcinoma with Lack of Response to Afatinib. *J Thorac Oncol* 2017; 12: e16-e18. 2017/01/25. DOI: 10.1016/j.jtho.2016.11.273.

50. Yang X, Chen H, Zhang H, et al. Effectiveness of Tyrosine Kinase Inhibitors on Uncommon Epidermal Growth Factor Receptor Mutations in Non-small Cell Lung Cancer. *Zhongguo Fei Ai Za Zhi* 2015; 18: 493-499. 2015/08/25. DOI: 10.3779/j.issn.1009-3419.2015.08.04.

51. Del Re M, Rofi E, Cappelli C, et al. The increase in activating EGFR mutation in plasma is an early biomarker to monitor response to osimertinib: a case report. *BMC Cancer* 2019; 19: 410. 2019/05/02. DOI: 10.1186/s12885-019-5604-6.

52. Zhu N, Dong C, Weng S, et al. A Patient of Advanced NSCLC with a New EGFR Exon 19 Insertion Mutation and its Response to EGFR-TKIs. *J Coll Physicians Surg Pak* 2019; 29: S126-S128. DOI: 10.29271/jcpsp.2019.12.S126.

53. Lin L, Wu X, Yan S, et al. Response to Afatinib in a Patient with NSCLC Harboring Novel EGFR Exon 20 Insertion Mutations. *Onco Targets Ther* 2020; 13: 9753-9757. 2020/10/17. DOI: 10.2147/OTT.S268694.

54. Chen LC, Shih JY, Yu CJ, et al. A rare epidermal growth factor receptor H773L/V774M compound mutation in advanced non-small-cell lung cancer with poor response to epidermal growth factor receptor tyrosine kinase inhibitor. *Respirol Case Rep* 2019; 7: e00425. 2019/04/23. DOI: 10.1002/rcr2.425.

55. Zhang C, Lin L, Zuo R, et al. Response to tyrosine kinase inhibitors in lung adenocarcinoma with the rare epidermal growth factor receptor mutation S768I and G724S: A case report and literature review. *Thorac Cancer* 2020; 11: 2743-2748. 2020/08/11. DOI: 10.1111/1759-7714.13606.

56. Yamaguchi T, Hayashi H, Isogai S, et al. Afatinib administration in a patient with non-small cell lung cancer harboring uncommon EGFR mutation G719A undergoing hemodialysis. *Cancer Treatment Communications* 2015; 4: 169-171. DOI: 10.1016/j.ctrc.2015.09.006.

57. Li BT, Lee A, O'Toole S, et al. HER2 insertion YVMA mutant lung cancer: Long natural history and response to afatinib. *Lung Cancer* 2015; 90: 617-619. 2015/11/13. DOI: 10.1016/j.lungcan.2015.10.025.

58. Yang Y, Zhang X, Wang R, et al. Osimertinib Resistance With a Novel EGFR L858R/A859S/Y891D Triple Mutation in a Patient With Non-Small Cell Lung Cancer: A Case Report. *Front Oncol* 2020; 10: 542277. 2020/12/17. DOI: 10.3389/fonc.2020.542277.

59. Ma C, Liu M, Mu N, et al. Efficacy of afatinib for pulmonary adenocarcinoma with leptomeningeal metastases harboring an epidermal growth factor receptor complex mutation (exon 19del+K754E): A case report. *Medicine (Baltimore)* 2020; 99: e22851. 2020/10/31. DOI: 10.1097/MD.0000000000022851.

60. Long X, Qin T and Lin J. Great Efficacy of Afatinib in a Patient with Lung Adenocarcinoma Harboring EGFR L833V/H835L Mutations: A Case Report. *Onco Targets Ther* 2020; 13: 10689-10692. 2020/10/30. DOI: 10.2147/OTT.S260157.

61. Ma C, Wang S, Mu N, et al. Effective Treatment With Afatinib of Lung Adenocarcinoma With Leptomeningeal Metastasis Harboring the Exon 18 p.G719A Mutation in the EGFR Gene Was Detected in Cerebrospinal Fluid: A Case Report. *Front Oncol* 2020; 10: 1635. 2020/10/06. DOI: 10.3389/fonc.2020.01635.

62. Shen YC, Tseng GC, Tu CY, et al. Comparing the effects of afatinib with gefitinib or Erlotinib in patients with advanced-stage lung adenocarcinoma harboring non-classical epidermal growth factor receptor mutations. *Lung Cancer* 2017; 110: 56-62. 2017/07/06. DOI: 10.1016/j.lungcan.2017.06.007.

63. Tamiya M, Kunimasa K, Nishino K, et al. Successful treatment of an osimertinib-resistant lung adenocarcinoma with an exon 18 EGFR mutation (G719S) with afatinib plus bevacizumab. *Invest New Drugs* 2021; 39: 232-236. 2020/06/20. DOI: 10.1007/s10637-020-00966-7.

64. Urban L, Doczi R, Vodicska B, et al. Major Clinical Response to Afatinib Monotherapy in Lung Adenocarcinoma Harboring EGFR Exon 20 Insertion Mutation. *Clin Lung Cancer* 2021; 22: e112-e115. 2020/10/22. DOI: 10.1016/j.cllc.2020.09.005.

65. Minari R, Leonetti A, Gnetti L, et al. Afatinib therapy in case of EGFR G724S emergence as resistance mechanism to osimertinib. *Anticancer Drugs* 2021 2021/03/07. DOI: 10.1097/CAD.0000000000001064.

66. Morita A, Hosokawa S, Yamada K, et al. Dacomitinib as a retreatment for advanced non-small cell lung cancer patient with an uncommon EGFR mutation. *Thorac Cancer* 2021; 12: 1248-1251. 2021/03/03. DOI: 10.1111/1759-7714.13897.

67. Duan H, Peng Y, Cui H, et al. Effectiveness of afatinib after ineffectiveness of gefitinib in an advanced lung adenocarcinoma patient with a single EGFR exon 20 S768I mutation: a case report. *Onco Targets Ther* 2018; 11: 2303-2309. 2018/05/08. DOI: 10.2147/OTT.S151125.

68. Shan J, Ruan J, Tan Y, et al. Efficacy of Pyrotinib in a Heavily Pretreated Patient with Lung Adenocarcinoma Harboring HER2 Amplification and Exon 20 Insertions: A Case Report. *Onco Targets Ther* 2020; 13: 9849-9856. 2020/10/17. DOI: 10.2147/OTT.S271999.

69. Vasconcelos PENS, Gergis C, Viray H, et al. EGFR-A763_Y764insFQEA Is a Unique Exon 20 Insertion Mutation That Displays Sensitivity to Approved and In-Development Lung Cancer EGFR Tyrosine Kinase Inhibitors. *JTO Clinical and Research Reports* 2020; 1. DOI: 10.1016/j.jtocrr.2020.100051.

70. Sekine A, Katano T, Oda T, et al. Miliary lung metastases from non-small cell lung cancer with Exon 20 insertion: A dismal prognostic entity: A case report. *Mol Clin Oncol* 2018; 9: 673-676. 2018/12/14. DOI: 10.3892/mco.2018.1730.

71. Kosaka T, Tanizaki J, Paranal RM, et al. Response Heterogeneity of EGFR and HER2 Exon 20 Insertions to Covalent EGFR and HER2 Inhibitors. *Cancer Research* 2017; 77: 2712-2721. DOI: 10.1158/0008-5472.Can-16-3404.

72. Jelli B, Taton O, D'Haene N, et al. Complete Response to Afatinib of an EGFR Exon 18 delE709_T710insD-Mutated Stage IV Lung Adenocarcinoma. *Eur J Case Rep Intern Med* 2021; 8: 002749. 2021/09/17. DOI: 10.12890/2021_002749.

73. Van Acker L, Stevens D, Vermaelen K, et al. Afatinib for the treatment of advanced non-small-cell lung cancer harboring an epidermal growth factor receptor exon 18 E709_T710delinsD mutation: a case report. *J Med Case Rep* 2021; 15: 562. 2021/11/24. DOI: 10.1186/s13256-021-02994-0.

74. Bi H, Ren D, Wu J, et al. Lung squamous cell carcinoma with rare epidermal growth factor receptor mutation G719X: a case report and literature review. *Ann Transl Med* 2021; 9: 1805. 2022/01/25. DOI: 10.21037/atm-21-6653.

75. Wei Y, Cui Y, Guo Y, et al. A Lung Adenocarcinoma Patient With a Rare EGFR E709_T710delinsD Mutation Showed a Good Response to Afatinib Treatment: A Case Report and Literature Review. *Front Oncol* 2021; 11: 700345. 2021/06/29. DOI: 10.3389/fonc.2021.700345.

76. Liu X, Ma B, Li T, et al. Case Report: Afatinib-Induced Interstitial Pneumonia: Experiences and Lessons From Two Patients. *Front Pharmacol* 2021; 12: 698447. 2021/11/02. DOI: 10.3389/fphar.2021.698447.

77. Huang Q, Chen C, Hu S, et al. Long-term survival in a patient with advanced lung adenocarcinoma harboring synchronous EGFR exon 18 G719A and BRAF V600E mutations and treated with afatinib: a case report. *Anticancer Drugs* 2022; 33: e730-e733. 2021/08/14. DOI: 10.1097/CAD.0000000000001159.

78. Gow CH, Liao WY, Liu YN, et al. Discordant HER2 Exon 20 Mutation Status Determines a Differential Sensitivity to Afatinib. *J Thorac Oncol* 2015; 10: e58-60. 2015/07/03. DOI: 10.1097/JTO.0000000000000518.

79. Xiang C, Zhang W, Xiong LW, et al. EGFR Thr790Leu as a Potential Resistance Mechanism to First-Generation EGFR Tyrosine Kinase Inhibitor May Respond to Osimertinib in Patients With Lung Adenocarcinoma. *JTO Clin Res Rep* 2021; 2: 100185. 2021/10/01. DOI: 10.1016/j.jtocrr.2021.100185.

80. Qu F, Wu S, Dong H, et al. An elderly advanced non-small cell lung cancer patient harboring rare epidermal growth factor receptor mutations L861R benefited from afatinib: A case report. *Medicine (Baltimore)* 2021; 100: e27614. 2021/11/13. DOI: 10.1097/MD.0000000000027614.

81. Yu N, Xu Y, Wang X, et al. Successful Treatment of Afatinib Reversing Epidermal Growth Factor Receptor Exon19Deletion/G724S Mutation Resistance Guided by Protein-Drug Docking. *Oncologist* 2021; 26: e1903-e1908. 2021/08/17. DOI: 10.1002/onco.13932.

82. Longo V, Catino A, Montrone M, et al. Successful treatment of triple EGFR mutation T785A/L861Q/H297_E298 with afatinib. *Thorac Cancer* 2021; 12: 2031-2034. 2021/05/20. DOI: 10.1111/1759-7714.13953.

83. Ohara G, Okauchi S, Sasatani Y, et al. Long-term Survival With Afatinib in a Patient With Lung Adenocarcinoma Harboring Double Uncommon EGFR L861Q and G719X Mutations. *In Vivo* 2020; 34: 1459-1462. 2020/05/02. DOI: 10.21873/invivo.11929.

84. Zhang Y, Shen JQ, Shao L, et al. Non-small-cell lung cancer with epidermal growth factor receptor L861Q-L833F compound mutation benefits from both afatinib and osimertinib: A case report. *World J Clin Cases* 2021; 9: 8220-8225. 2021/10/09. DOI: 10.12998/wjcc.v9.i27.8220.

85. Guo T, Zhu L, Li W, et al. Two cases of non-small cell lung cancer patients with somatic or germline EGFR R776H mutation. *Lung Cancer* 2021; 161: 94-97. 2021/09/24. DOI: 10.1016/j.lungcan.2021.05.036.

86. Zhao Y, Zhai L, Deng L, et al. Efficacy of Osimertinib in Afatinib-resistant Lung Cancer Harboring Uncommon EGFR Mutations: Case Report and Literature Review. *Clin Lung Cancer* 2021; 22: e466-e469. 2020/07/23. DOI: 10.1016/j.cllc.2020.06.017.

87. Fang W, Huang Y, Hong S, et al. EGFR exon 20 insertion mutations and response to osimertinib in non-small-cell lung cancer. *BMC Cancer* 2019; 19: 595. 2019/06/19. DOI: 10.1186/s12885-019-5820-0.

88. Peng X, Long X, Liu L, et al. Clinical impact of uncommon epidermal growth factor receptor exon 19 insertion-deletion variants on epidermal growth factor receptor-tyrosine kinase inhibitor efficacy in non-small-cell lung cancer. *Eur J Cancer* 2020; 141: 199-208. 2020/11/11. DOI: 10.1016/j.ejca.2020.10.005.

89. Zhang L, Yang X, Ming Z, et al. Molecular Characteristics of the Uncommon EGFR Exon 21 T854A Mutation and Response to Osimertinib in Patients With Non-Small Cell Lung Cancer. *Clin Lung Cancer* 2021 2022/01/21. DOI: 10.1016/j.cllc.2021.12.008.

90. Okuno T, Arakawa S, Yoshida T, et al. Efficacy of osimertinib in a patient with leptomeningeal metastasis and EGFR uncommon S768I mutation. *Lung Cancer* 2020; 143: 95-96. 2020/03/27. DOI: 10.1016/j.lungcan.2020.03.016.

91. Simionato F, Calvetti L, Cosci M, et al. Case Report: A Metabolic Complete Response to Upfront Osimertinib in a Smoker Non-Small Cell Lung Cancer Patient Harbouring EGFR G719A/V769M Complex Mutation. *Onco Targets Ther* 2020; 13: 12027-12031. 2020/12/03. DOI: 10.2147/OTT.S280933.

92. Huang X, Yang Y, Wang P, et al. A rare EGFR mutation L747P conferred therapeutic efficacy to both ge fi tinib and osimertinib: A case report. *Lung Cancer* 2020; 150: 9-11. 2020/10/10. DOI: 10.1016/j.lungcan.2020.09.017.

93. Zhu Y, Tang J, Li X, et al. Durable Response to Osimertinib in a Chinese Patient with Metastatic Lung Adenocarcinoma Harboring a Rare EGFR L858R/D761Y Compound Mutation. *Onco Targets Ther* 2020; 13: 10447-10451. 2020/10/30. DOI: 10.2147/OTT.S268593.

94. Inagaki Y, Tamiya A, Matsuda Y, et al. Poor effect of osimertinib on EGFR exon 20 insertion-positive lung adenocarcinoma: A case report. *Medicine (Baltimore)* 2020; 99: e22628. 2020/10/22. DOI: 10.1097/MD.0000000000022628.

95. Li H, Yu T, Lin Y, et al. Three Novel EGFR Mutations (750_758del, I759S, T751_I759delinsS) in One Patient with Metastatic Non-Small Cell Lung Cancer Responding to Osimertinib: A Case Report. *Onco Targets Ther* 2020; 13: 7941-7948. 2020/09/29. DOI: 10.2147/OTT.S259616.

96. Ikari T, Sakakibara-Konishi J, Yamamoto G, et al. Response to First-Line Osimertinib Treatment in Non-Small-Cell Lung Cancer With Coexisting G719A and Primary T790M Epidermal Growth Factor Receptor Mutations. *Clin Lung Cancer* 2019; 20: e531-e533. 2019/06/06. DOI: 10.1016/j.cllc.2019.05.002.

97. Yang M, Tong X, Xu X, et al. Case Report: Osimertinib achieved remarkable and sustained disease control in an advanced non-small-cell lung cancer harboring EGFR H773L/V774M mutation complex. *Lung Cancer* 2018; 121: 1-4. 2018/06/03. DOI: 10.1016/j.lungcan.2018.04.006.

98. Huang J, Wang Y, Zhai Y, et al. Non-small cell lung cancer harboring a rare EGFR L747P mutation showing intrinsic resistance to both gefitinib and osimertinib (AZD9291): A case report. *Thorac Cancer* 2018; 9: 745-749. 2018/04/20. DOI: 10.1111/1759-7714.12637.

99. Huang X, Yang Y, Wang P, et al. A heavily pre-treated adenocarcinoma patient with EGFR exon 20 insertion mutation responded to pembrolizumab plus nab-paclitaxel/bevacizumab: a case report. *Ann Palliat Med* 2021; 10: 6997-7002. 2020/11/14. DOI: 10.21037/apm-20-1307.

100. Fang W, Huang Y, Gan J, et al. A Patient with EGFR Exon 20 Insertion-Mutant Non-Small Cell Lung Cancer Responded to Osimertinib plus Cetuximab Combination Therapy. *J Thorac Oncol* 2019; 14: e201-e202. 2019/08/26. DOI: 10.1016/j.jtho.2019.04.013.

101. Hakozaki T and Yomota M. Acquisition of T790M resistance mutation in a patient with advanced adenocarcinoma harbouring uncommon EGFR mutations: a case report and literature review. *Onco Targets Ther* 2019; 12: 745-748. 2019/02/19. DOI: 10.2147/OTT.S190034.

102. Grolleau E, Haddad V, Boissiere L, et al. Clinical Efficacy of Osimertinib in a Patient Presenting a Double EGFR L747S and G719C Mutation. *J Thorac Oncol* 2019; 14: e151-e153. 2019/06/27. DOI: 10.1016/j.jtho.2019.02.034.

103. Coleman N, Woolf D, Welsh L, et al. EGFR Exon 20 Insertion (A763_Y764insFQEA) Mutant NSCLC Is Not Identified by Roche Cobas Version 2 Tissue Testing but Has Durable Intracranial and Extracranial Response to Osimertinib. *J Thorac Oncol* 2020; 15: e162-e165. 2020/09/29. DOI: 10.1016/j.jtho.2020.05.006.

104. Zhang Q, Jiang T, Xiao M, et al. Identification of a Novel Osimertinib-Sensitive Mutation, EGFR H773L, in a Chinese Patient With NSCLC. *J Thorac Oncol* 2020; 15: e46-e48. 2020/02/26. DOI: 10.1016/j.jtho.2019.11.018.

105. Kunimasa K, Nishino K, Kukita Y, et al. Late recurrence of lung adenocarcinoma harboring EGFR exon 20 insertion (A763_Y764insFQEA) mutation successfully treated with osimertinib. *Cancer Genet* 2021; 256-257: 57-61. 2021/04/27. DOI: 10.1016/j.cancergen.2021.04.001.

106. Onozawa H, Saito H, Sunami K, et al. Lung adenocarcinoma in a patient with a cis EGFR L858R-K860I doublet mutation identified using NGS-based profiling test: Negative diagnosis on initial companion test and successful treatment with osimertinib. *Thorac Cancer* 2020; 11: 3599-3604. 2020/10/10. DOI: 10.1111/1759-7714.13694.

107. Murano C, Igarashi A, Yamauchi K, et al. Osimertinib as treatment for EGFR exon 20 insertion-positive lung adenocarcinoma. *EXCLI J* 2019; 18: 893-898. 2019/10/28. DOI: 10.17179/excli2019-1786.

108. Song H, Chen Y, Yan Z, et al. Response to Osimertinib in a NSCLC Patient Harboring EGFR V843I Germ-Line Mutation. *Lung Cancer* 2020; 150: 247-248. 2020/10/18. DOI: 10.1016/j.lungcan.2020.09.019.

109. Piotrowska Z, Fintelmann FJ, Sequist LV, et al. Response to Osimertinib in an EGFR Exon 20 Insertion-Positive Lung Adenocarcinoma. *J Thorac Oncol* 2018; 13: e204-e206. 2018/09/25. DOI: 10.1016/j.jtho.2018.05.017.

110. Shan CG, Wang H, Lin T, et al. A non-small cell lung cancer (NSCLC) patient with leptomeningeal metastasis harboring rare epidermal growth factor receptor (EGFR) mutations G719S and L861Q benefited from doubling dosage of osimertinib: a case report. *Ann Palliat Med* 2021; 10: 5897-5901. 2021/05/13. DOI: 10.21037/apm-20-2556.

111. Sehgal K, Rangachari D, VanderLaan PA, et al. Clinical Benefit of Tyrosine Kinase Inhibitors in Advanced Lung Cancer with EGFR-G719A and Other Uncommon EGFR Mutations. *Oncologist* 2021; 26: 281-287. 2020/09/25. DOI: 10.1002/onco.13537.

112. Cai Y, Wang Y, Sun J, et al. Successful treatment of a patient with NSCLC carrying uncommon compound EGFR G719X and S768I mutations using osimertinib: A case report. *J Int Med Res* 2020; 48: 300060520928793. 2020/06/05. DOI: 10.1177/0300060520928793.

113. Ito N, Masuda T, Ooka I, et al. First-line osimertinib treatment in a patient with lung adenocarcinoma with coexisting epidermal growth factor receptor G719S and de novo T790M mutations. *Thorac Cancer* 2022 2022/01/26. DOI: 10.1111/1759-7714.14288.

114. Wang Y, Liu S, Feng A, et al. Identification of a Rare EGFR T790I Mutation in Lung Adenocarcinoma Sensitive to Osimertinib. *Front Oncol* 2021; 11: 727312. 2021/11/09. DOI: 10.3389/fonc.2021.727312.

115. Lin R, Chen R, Chen Z, et al. Efficacy of Osimertinib in NSCLC Harboring Uncommon EGFR L861Q and Concurrent Mutations: Case Report and Literature Review. *Front Oncol* 2021; 11: 731572. 2021/09/21. DOI: 10.3389/fonc.2021.731572.

116. Zhi X, Luo J, Li W, et al. Case Report: Osimertinib Followed by Osimertinib Plus Bevacizumab, Personalized Treatment Strategy for a Lung Cancer Patient With a Novel EGFR Exon 20 Insertion D770_N771insGT and Multiple Brain Metastases. *Front Oncol* 2021; 11: 733276. 2021/11/12. DOI: 10.3389/fonc.2021.733276.

117. Fang YF and Liu PC. Afatinib and osimertinib in lung adenocarcinoma harbored EGFR T751_I759delinsS mutation: A case report. *Thorac Cancer* 2021; 12: 3429-3432. 2021/11/04. DOI: 10.1111/1759-7714.14215.

Supplementary Table 2 Baseline characteristics before PSM

| **Characteristics** | Total (*N*=196) | AFA (*N*=125) | OSI (*N*=71) | *P* |
| --- | --- | --- | --- | --- |
| **Age** |  |  |  |  |
| median, years (range) | 59(25-84) | 58(26-84) | 61(25-83) |  |
| < 65 | 125 | 81 | 44 | 0.692 |
| ≥ 65 | 71 | 44 | 27 |  |
| **Gender** |  |  |  |  |
| Male | 93 | 65 | 28 | 0.090 |
| Female | 103 | 60 | 43 |  |
| **Ethnicity** |  |  |  |  |
| Asian | 146 | 81 | 65 | **< 0.001** |
| Non-Asian | 50 | 44 | 6 |  |
| **Smoking** |  |  |  |  |
| Yes | 69 | 46 | 23 | 0.457 |
| No | 111 | 71 | 40 |  |
| N/A | 16 | 8 | 8 |  |
| **Stage** |  |  |  |  |
| III | 9 | 5 | 4 | 0.599 |
| Ⅳ | 187 | 120 | 67 |  |
| **BM before AFA/OSI** |  |  |  |  |
| Yes | 57 | 26 | 31 | **< 0.001** |
| No | 93 | 59 | 34 |  |
| N/A | 46 | 40 | 6 |  |
| **Mutation types** |  |  |  |  |
| Single | 117 | 85 | 32 | **0.002** |
| Compound | 79 | 40 | 39 |  |
| **AFA/OSI line** |  |  |  |  |
| 1 L | 80 | 53 | 27 | 0.549 |
| ≥ 2 L | 116 | 72 | 44 |  |

Abbreviations: PSM: propensity score matching; BM: brain metastases; AFA: afatinib; OSI: osimertinib; N/A: not available

Supplementary Table 3 Clinical benefit according major clinical features

| **Characteristics** | No. of patients | Tumor response | |  | Survival | |
| --- | --- | --- | --- | --- | --- | --- |
|  |  | ORR | *P* |  | PFS (95%CI) | *P* |
| **AFA/OSI line** |  |  | **< 0.001** |  |  | **< 0.001** |
| 1 L | 80 | 70.0% |  |  | 15.0 (10.7-19.3) |  |
| ≥ 2 L | 116 | 43.1% |  |  | 6.0 (5.0-7.0) |  |
| **Mutation types** |  |  | **0.049** |  |  | 0.116 |
| Single | 117 | 59.8% |  |  | 10.0 (7.1-12.9) |  |
| Compound | 79 | 45.6% |  |  | 7.7 (5.6-9.8) |  |
| **Molecular subtypes** |  |  | 0.679 |  |  | **0.034** |
| Exon 20ins | 41 | 51.2% |  |  | 5.1 (2.8-7.4) |  |
| Non-exon 20ins | 155 | 54.8% |  |  | 10.0 (7.8-12.2) |  |

Abbreviations: BM: brain metastases; AFA: afatinib; OSI: osimertinib;

Supplementary Table 4 Baseline characteristics of subgroups

| **Characteristics** | Group A  (N=23) | Group B  (N=46) | Group C  (N=39) | Group D  (N=34) |
| --- | --- | --- | --- | --- |
| **Age** |  |  |  |  |
| median (range) | 59(25-84) | 58(26-84) | 61(25-83) | 65(36-81) |
| **Gender** |  |  |  |  |
| Male | 4(17.4%) | 14(30.4%) | 19(48.7%) | 21(61.8%) |
| Female | 19(82.6%) | 32(69.6%) | 20(51.3) | 13(38.2%) |
| **Ethnicity** |  |  |  |  |
| Asian | 21(91.3%) | 40(87.0%) | 34(87.2%) | 30(88.2%) |
| Non-Asian | 2(8.7%) | 6(13.0%) | 5(12.8%) | 4(11.8%) |
| **Smoking** |  |  |  |  |
| Yes | 5(21.7%) | 10(21.7%) | 12(30.8%) | 16(47.1%) |
| No | 18(78.3%) | 28(60.9%) | 22(56.4%) | 15(44.1%) |
| N/A | 0(0.0%) | 8(17.4%) | 5(12.8%) | 3(8.8%) |
| **Stage** |  |  |  |  |
| III | 0(0.0%) | 3(6.5%) | 3(7.7%) | 1(2.9%) |
| Ⅳ | 23(100.0%) | 43(93.5%) | 36(92.3%) | 33(97.1%) |
| **BM before AFA/OSI** |  |  |  |  |
| Yes | 9(39.1%) | 17(37.05) | 14(35.9%) | 14(41.2%) |
| No | 14(60.9%) | 28(60.9%) | 14(35.95) | 15(44.1%) |
| N/A | 0(0.0%) | 1(2.2%) | 11(28.2%) | 5(14.7%) |
| **EGFR-TKI** |  |  |  |  |
| OSI | 16(69.5%) | 16(34.8%) | 25(64.1%) | 14(41.2%) |
| AFA | 7(30.4%) | 30(65.2%) | 14(35.9%) | 20(58.8%) |
| **AFA/OSI line** |  |  |  |  |
| 1 Line | 9(39.1%) | 27(58.7%) | 7(17.9%) | 16(47.1%) |
| ≥ 2 Line | 14(60.9%) | 19(41.3%) | 32(82.1%) | 18(52.9%) |

Abbreviations: BM: brain metastases; AFA: afatinib; OSI: osimertinib; N/A: not available
