# Supplementary material for: Clinical Outcomes of Afatinib Versus Osimertinib in Patients With Non-Small Cell Lung Cancer With Uncommon EGFR Mutations: A Pooled Analysis
Source: Oncologist. 2023 Apr 28;28(6):e397–405. doi: 10.1093/oncolo/oyad111 (PMC10243768; doi:10.1093/oncolo/oyad111)
Supplement: oyad111_suppl_Supplementary_Figure_Captions [file oyad111_suppl_supplementary_figure_captions.docx]

**Supplementary Figure Legends**

**Supplementary Figure 1.** Composition of uncommon EGFR mutations after propensity score matching (n=142), mutations with lower frequencies are listed below, each of which has only one patient, accounting for 0.7%.

**Supplementary Figure 2.** Comparison of treatment response and progression free survival (PFS) between afatinib versus osimertinib in patients with non-exon 20 insertions (osimertinib group set as the reference group). BM: brain metastases; AFA: afatinib; OSI: osimertinib.
